# Clinical Utility and Genomic Landscape of Comprehensive Genomic Profiling in Biliary Tract Tumors: A Single-Center Real-World Study

**DOI:** 10.3390/cancers18142189

**Published:** 2026-07-08

**Authors:** Kazunori Nakaoka, Hiroyuki Kato, Seiji Yamada, Fumino Kato, Yusaku Urakawa, Gakushi Koumura, Hiroyuki Tanaka, Takuji Nakano, Sayaka Ueno, Teiji Kuzuya, Hiroshi Matsuoka, Tamotsu Sudo, Yoshiki Hirooka, Eizaburo Ohno

**Affiliations:** 1 Department of Gastroenterology, Fujita Health University School of Medicine, Toyoake 470-1192, Japan; knakaoka@fujita-hu.ac.jp (K.N.); gakushi.komura@fujita-hu.ac.jp (G.K.); hiroyuki.tanaka@fujita-hu.ac.jp (H.T.); tkjnkn@fujita-hu.ac.jp (T.N.); teiji.kuzuya@fujita-hu.ac.jp (T.K.); yoshiki.hirooka@fujita-hu.ac.jp (Y.H.); 2 Department of Gastroenterological Surgery, Fujita Health University School of Medicine Bantane Hospital, Nagoya 454-8509, Japan; katohiroyuki510719@gmail.com; 3 Division of Analytical Pathology, Oncology Invitation Center, Fujita Health University School of Medicine, Toyoake 470-1192, Japan; yamadas@fujita-hu.ac.jp; 4 Department of Cancer Genetics and Genomics, Fujita Health University Hospital, Toyoake 470-1192, Japan; fumino.kato@fujita-hu.ac.jp (F.K.); yusaku.urakawa@fujita-hu.ac.jp (Y.U.); sayaka007007@gmail.com (S.U.); tamotsu.sudo@fujita-hu.ac.jp (T.S.); 5 Department of Surgery, Fujita Health University School of Medicine, Toyoake 470-1192, Japan; mats1025@fujita-hu.ac.jp

**Keywords:** biliary tract tumor, comprehensive genomic profiling, precision oncology, actionable alteration, genomically matched therapy, genomic landscape

## Abstract

**Background:** Comprehensive genomic profiling (CGP) is increasingly incorporated into the management of biliary tract tumors (BTTs); however, single-center real-world data integrating clinical utility and site-specific genomic landscape remain limited. **Methods:** This retrospective single-center observational study included 91 patients with BTTs who underwent CGP. The primary endpoints were the rates of potentially actionable genomic alterations, expert panel-based therapeutic options, and implementation of genomically matched therapy. Secondary endpoints included the distribution of recurrent and clinically relevant genomic alterations according to the primary tumor site. **Results:** The cohort included 46 patients with intrahepatic cholangiocarcinoma (iCCA), 19 with extrahepatic cholangiocarcinoma (eCCA), 24 with gallbladder cancer (GBC), and 2 with ampullary tumors. CGP was successfully performed in all 91 patients. Reportable genomic alterations were detected in 88 patients (96.7%), whereas no reportable alteration was detected in three patients (3.3%). The most frequently altered genes were TP53 (51/91, 56.0%), KRAS (21/91, 23.1%), CDKN2A (18/91, 19.8%), SMAD4 (14/91, 15.4%), and ARID1A/PBRM1 (12/91, 13.2%). Potentially actionable genomic alterations were identified in 21 patients (23.1%). Expert panel-based therapeutic options were identified in 15 patients (16.5%), and genomically matched therapy was introduced in 7 patients (7.7%). Matched therapy was introduced the most frequently in iCCA (6/46, 13%), followed by GBC (1/24, 4.2%), whereas no patient with eCCA or ampullary tumor received matched therapy. **Conclusions:** CGP revealed a heterogeneous genomic landscape across BTT subtypes and provided clinically actionable information in a limited but meaningful subset of patients. The practical utility of CGP was the greatest in selected molecular subsets, particularly iCCA.

## Simple Summary

Biliary tract tumors are challenging cancers with diverse molecular characteristics. While comprehensive genomic profiling can identify genomic abnormalities that guide treatment selection, the extent to which these findings translate into actual treatment remains unclear. In this single-center study, genomic abnormalities that could be therapeutic targets were identified in 21 out of 91 biliary tract tumor patients, treatment options based on an expert panel were identified in 15 patients, and genomic information-based treatment was implemented in 7 patients. Treatment-related findings were most frequently observed in intrahepatic cholangiocarcinoma. These results highlight both the clinical value of comprehensive genomic profiling and the gap that still exists between genomic findings and actual treatment implementation.

## 1. Introduction

Biliary tract tumors (BTTs), including intrahepatic cholangiocarcinoma (iCCA), extrahepatic cholangiocarcinoma (eCCA), gallbladder cancer (GBC), and ampullary tumors, remain highly aggressive malignancies with poor long-term outcomes despite recent advances in systemic therapy. Although combination chemotherapy, immune checkpoint inhibitors, and selected molecularly targeted agents have expanded treatment options, the prognosis of advanced BTTs remains unsatisfactory. Accordingly, there is increasing interest in incorporating molecular information into routine clinical decision-making beyond conventional anatomical classification [1].

Accumulating genomic studies have shown that BTTs are a heterogeneous group of malignancies with distinct molecular characteristics according to the primary site rather than a biologically uniform disease entity. Nakamura et al. demonstrated that iCCA, eCCA, and GBC harbor different genomic alterations, thereby establishing a molecular basis for precision oncology in BTTs [1]. These findings indicate that comprehensive genomic profiling (CGP) may provide clinically meaningful information for treatment selection in a subset of patients. CGP has become increasingly important in the management of advanced solid tumors because it enables the simultaneous assessment of multiple genomic alterations that may inform treatment selection, clinical trial enrollment, and therapeutic stratification. However, the clinical utility of CGP in daily practice is influenced by the detection of actionable alterations and real-world factors such as test timing, specimen availability, patient condition, access to matched therapies, and institutional treatment pathways. In real-world clinical practice, the gap between the identification of potentially actionable alterations and the actual administration of genomically matched therapy remains an important problem [2]. Although several studies have reported the clinical significance of CGP in BTTs, real-world data focusing on single-center experience remain limited. A recent real-world study showed that CGP testing in advanced BTTs yielded treatment recommendations in a substantial proportion of patients and enabled genomically matched therapy in a smaller but clinically meaningful subset, particularly in iCCA [3]. In parallel, large-scale database analyses have further clarified the biological and prognostic significance of specific genomic alterations in BTTs, including KRAS variants [4]. Moreover, the Pan-Asian-adapted European Society For Medical Oncology (ESMO) Clinical Practice Guidelines position molecularly guided therapy as an integral component of second- and later-line treatment for BTTs, underscoring the practical importance of identifying actionable alterations in real-world practice [5]. Nevertheless, few studies have simultaneously evaluated the real-world clinical utility of CGP and comprehensively visualized the genomic landscape of a single institutional BTT cohort. The added value of the present cohort lies not simply in describing genomic alteration frequencies but in linking CGP findings to expert panel-based therapeutic options and actual implementation of genomically matched therapy in routine clinical practice.

Therefore, the aim of the present study was to evaluate the clinical utility of CGP in patients with BTTs treated at our institution and characterize the genomic landscape of this cohort according to the primary tumor site. Specifically, we investigated the frequency of clinically relevant and actionable genomic alterations, the rate of expert panel-based therapeutic recommendations, and the proportion of patients who ultimately received genomically matched therapy. In addition, we sought to visualize recurrent genomic alterations and co-alteration patterns across the cohort using an OncoPrint-style approach, thereby providing a clinically interpretable overview of the molecular features of BTTs in real-world practice [3,4].

## 2. Materials and Methods

### 2.1. Study Design and Patients

This retrospective single-center observational study included patients with BTTs who underwent CGP at our institution between September 2019 and April 2026. Eligible tumor types included iCCA, eCCA, GBC, and ampullary tumors. Patients with unavailable CGP reports, insufficient clinical information, or uncertain primary tumor origin were excluded.

### 2.2. Ethical Considerations

This study was approved by the Ethics Committee of Fujita Health University (Approval No.: HM22-375; approval date: 20 June 2016) and registered in the University Hospital Medical Information Network Clinical Trials Registry (Registration No.: UMIN000043526). This investigation was conducted in accordance with the principles stipulated in the Declaration of Helsinki.

### 2.3. Data Collection and Definitions

Clinical data were collected from medical records and the working spreadsheet, including age, sex, primary tumor site, clinical setting at the time of CGP, specimen type, CGP platform, prior treatment history, expert panel recommendations, and subsequent treatment course. Genomic data were extracted from the CGP reports. The CGP platforms used in the present study included FoundationOne CDx genome profiling (F1CDx; Chugai Pharmaceutical, Tokyo, Japan), FoundationOne Liquid CDx genome profiling (F1LCDx; Chugai Pharmaceutical), OncoGuide NCC Oncopanel System (NCC Oncopanel; Sysmex Corporation, Kobe, Japan), Guardant360 CDx (Guardant Health, Palo Alto, CA, USA), and GenMineTOP cancer genomic profiling system (GenMine Labs, Tokyo, Japan).

These platforms differ in specimen type and analytical scope; therefore, their major characteristics were described briefly. F1CDx is a tissue-based assay using formalin-fixed paraffin-embedded tumor tissue and analyzes 324 genes as well as selected genomic signatures, whereas F1LCDx analyzes 324 genes using circulating cell-free DNA isolated from plasma. The NCC Oncopanel is a tumor–normal paired tissue-based assay that detects mutations and copy number alterations in 124 genes, fusions in 13 genes, and microsatellite instability. Guardant360 CDx is a plasma-based next-generation sequencing assay for circulating tumor DNA. GenMineTOP is a tumor–normal paired CGP system using tumor tissue DNA/RNA and matched blood DNA, enabling broad detection of single-nucleotide variants, insertions/deletions, copy number alterations, gene fusions, exon-skipping events, and gene expression alterations. In routine clinical practice, the choice of CGP platform for each patient was determined based on the availability and suitability of tumor tissue, specimen quality, the timing of clinical implementation, and the testing systems available within the Japanese healthcare framework at the time of testing. When sufficient formalin-fixed, paraffin-embedded (FFPE) tumor tissue was available, tissue-based CGP was generally prioritized. Conversely, plasma-based CGP was selected when tumor tissue was insufficient or difficult to obtain or when liquid biopsy was deemed clinically appropriate.

Gene-level frequencies were calculated at the patient level; when multiple alterations in the same gene were reported in a single patient, the gene was counted once for frequency analysis. Clinically relevant or potentially actionable genomic alterations and biomarkers included fibroblast growth factor receptor 2 (FGFR2) fusions or rearrangements, isocitrate dehydrogenase 1 (IDH1) mutations, erb-b2 receptor tyrosine kinase 2 (ERBB2) alterations, B-Raf proto-oncogene, serine/threonine kinase (BRAF) V600E, microsatellite instability-high (MSI-H) status, DNA damage repair-related alterations such as breast cancer gene 1/2 (BRCA1/2), partner and localizer of BRCA2 (PALB2), and ataxia telangiectasia mutated (ATM) alterations, and MET proto-oncogene, receptor tyrosine kinase (MET) amplification. The clinical significance and actionability of genomic alterations were interpreted with reference to professional guidelines for the interpretation and reporting of sequence variants in cancer, including the joint consensus recommendations of the Association for Molecular Pathology, American Society of Clinical Oncology, and College of American Pathologists, as well as literature review and expert consensus through an institutional expert panel [6]. In contrast, recurrent alterations such as KRAS, tumor protein p53 (TP53), cyclin dependent kinase inhibitor 2A/B (CDKN2A/B), methylthioadenosine phosphorylase (MTAP), and SMAD4 were treated primarily as biologically relevant genomic findings unless a realistic therapeutic option was identified through expert panel discussion.

Expert panel-based therapeutic options were defined as approved agents, off-label molecularly targeted therapies, immune checkpoint inhibitors for biomarker-defined indications, or clinical trials judged to be relevant based on the CGP result and expert panel discussion. Genomically matched therapy was defined as systemic treatment introduced on the basis of a CGP finding and/or expert panel recommendation. In liquid CGP, ctDNA tumor fraction or an equivalent shedding-related index was considered an important interpretive parameter reflecting assay informativeness.

### 2.4. Study Endpoints and Post-CGP Outcome Assessment

The primary endpoints of this study were the rate of potentially actionable genomic alterations, the rate of expert panel-based therapeutic recommendations, and the rate of implementation of genomically matched therapy. Secondary endpoints included the distribution of clinically relevant genomic alterations according to the primary tumor site, visualization of recurrent genomic alterations and co-alteration patterns, and descriptive characterization of the post-CGP clinical course in patients who received genomically matched therapy. Survival outcomes were not evaluated in the present study.

### 2.5. Statistical Analysis

Categorical variables were summarized as numbers and percentages. Exact 95% confidence intervals (CIs) for major proportions were calculated using the Clopper–Pearson method. Exploratory comparisons between selected tumor-site subgroups and the remaining tumors were performed using Fisher’s exact test and are summarized in Supplementary Table S1. Because of the limited sample size, particularly in eCCA and ampullary tumors, these statistical comparisons were considered exploratory. No adjustment for multiple testing was performed.

## 3. Results

### 3.1. Patient Characteristics

The overall study cohort comprised 91 patients with BTTs. The median age was 66 years (range: 28–84 years), and more than half (57.1%) were male. Primary tumor sites included iCCA in 46 patients (50.5%), eCCA in 19 patients (20.9%), GBC in 24 patients (26.4%), and ampullary tumor in two patients (2.2%). At the time of CGP, 27 patients (29.7%) had postoperative recurrence and 64 patients (70.3%) had unresectable advanced disease. Tissue-based CGP was performed using F1CDx in 36 patients (39.6%), NCC Oncopanel in 32 patients (35.2%), and GenMineTOP in 10 patients (11.0%). Liquid CGP was performed using F1LCDx in five patients (5.5%) and Guardant360 CDx in eight patients (8.8%). Patient characteristics are summarized in Table 1.

### 3.2. Overall Genomic Findings

CGP was successfully performed in all 91 patients, and all cases were evaluable for genomic findings. Reportable genomic alterations were detected in 88 patients (96.7%), whereas no reportable genomic alteration was detected in the remaining three patients (3.3%). Thus, cases recorded as having no variant represented successfully tested cases without a detected reportable alteration rather than those with missing genomic data. The most commonly altered genes were TP53 (51/91, 56.0%), KRAS (21/91, 23.1%), CDKN2A (18/91, 19.8%), SMAD4 (14/91, 15.4%), AT-rich interaction domain 1A (ARID1A) (12/91, 13.2%), and polybromo 1 (PBRM1) (12/91, 13.2%). Other recurrent or clinically relevant alterations included IDH1 (8/91, 8.8%), ATM (7/91, 7.7%), adenomatosis polyposis coli (APC) (7/91, 7.7%), phosphatidylinositol-4,5-bisphosphate 3-kinase catalytic subunit alpha (PIK3CA) (7/91, 7.7%), CDKN2B (7/91, 7.7%), FGFR2 (6/91, 6.6%), MTAP (6/91, 6.6%), ERBB2 (5/91, 5.5%), and BRAF (5/91, 5.5%). MSI-H was recorded in five patients (5.5%). These findings demonstrate that the overall genomic landscape was dominated by TP53 and KRAS alterations, whereas therapeutically relevant alterations were distributed in site-specific patterns. The site-specific clinical utility of CGP is summarized in Table 2.

### 3.3. Potential Clinical Utility of CGP

Potentially actionable genomic alterations, according to the predefined study definition, were identified in 21 patients (23.1%). These findings included FGFR2 fusions/rearrangements, IDH1 mutations, ERBB2 alterations, BRAF V600E, BRCA1/2 or PALB2 alterations, ATM alterations, MET amplification, and MSI-H. In contrast, recurrent alterations, such as KRAS, FGFR3, CDKN2A/B, MTAP, and mouse double minute 2 were not classified as actionable in the present analysis; instead, they were treated as recurrent or biologically relevant genomic findings. Site-specific frequencies of potentially actionable alterations were highest in iCCA (17/46, 37.0%), followed by GBC (4/24, 16.7%), whereas no potentially actionable genomic alteration was identified in eCCA or ampullary tumors using this definition. Expert panel-based therapeutic options, including clinical trials and biomarker-guided systemic therapies, were identified in 15 patients (16.5%). Site-specific therapeutic options were most frequent in iCCA (11/46, 23.9%), followed by GBC (3/24, 12.5%) and eCCA (1/19, 5.3%), whereas no such option was identified in ampullary tumors. Actual treatment introduction was confirmed in 7 of 91 patients (7.7%), including 6 patients with iCCA (13.0%) and 1 patient with GBC (4.2%). No patient with eCCA or ampullary tumor received genomically matched therapy. These findings indicate that the real-world clinical benefit of CGP depends on the detection of potentially actionable genomic alterations, as well as the accessibility and timely introduction of matched therapies. The seven patients who received genomically matched therapy included those treated for MSI-H, FGFR2 fusion/rearrangement, ATM alteration, or BRCA2 alteration. In iCCA, matched treatment included pembrolizumab for MSI-H, FGFR inhibitors for FGFR2 fusion/rearrangement-positive tumors, and BAY 1895344, an ataxia telangiectasia and Rad3-related (ATR) inhibitor, administered through enrollment in a clinical trial for an ATM-altered tumor. In GBC, one patient with a BRCA2 alteration received a poly(ADP-ribose) polymerase (PARP) inhibitor through enrollment in a clinical trial. These cases are summarized in Table 3. In addition, subsequent confirmatory germline evaluation identified hereditary cancer predisposition in two patients: one with hereditary breast and ovarian cancer syndrome and one with Lynch syndrome. These findings indicate that CGP may also provide clinically meaningful clues to hereditary cancer predisposition in a small subset of patients with BTTs.

### 3.4. Site-Specific Genomic Patterns

**Figure 1:**
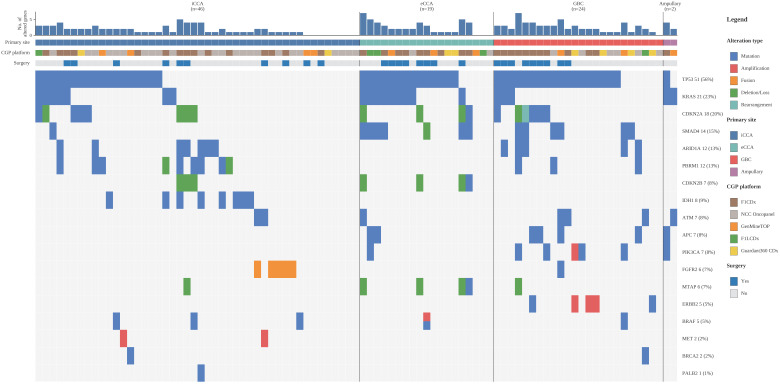
OncoPrint-style visualization of recurrent and clinically relevant genomic alterations across the cohort.

**Figure 2:**
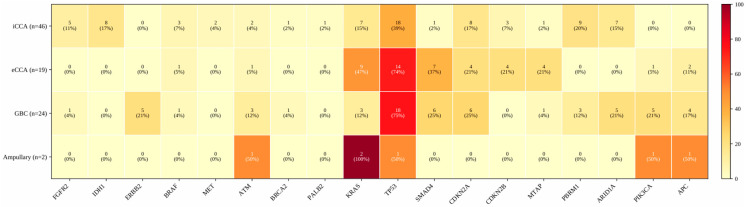
Site-specific frequency of selected clinically relevant genomic alterations.

The distribution of recurrent and clinically relevant genomic alterations differed according to the primary tumor site. In iCCA, the most frequent alterations were TP53 (18/46, 39.1%), PBRM1 (9/46, 19.6%), CDKN2A (8/46, 17.4%), IDH1 (8/46, 17.4%), KRAS (7/46, 15.2%), ARID1A (7/46, 15.2%), and FGFR2 (5/46, 10.9%). FGFR2 alterations in iCCA were mainly represented by fusions or rearrangements, and IDH1 alterations were confined to iCCA in this cohort. Less frequent but therapeutically relevant findings, including MET amplification and ATM alterations, were also observed in iCCA. In GBC, TP53 alterations were frequent (18/24, 75.0%), followed by CDKN2A (6/24, 25.0%), SMAD4 (6/24, 25.0%), ARID1A (5/24, 20.8%), ERBB2 (5/24, 20.8%), and PIK3CA (5/24, 20.8%). ERBB2 alterations were predominantly observed in GBC, supporting the molecular distinctiveness of this subgroup. In contrast, eCCA was characterized mainly by recurrent alterations rather than predefined potentially actionable alterations; the most frequent alterations were TP53 (14/19, 73.7%), KRAS (9/19, 47.4%), SMAD4 (7/19, 36.8%), CDKN2A (4/19, 21.1%), CDKN2B (4/19, 21.1%), and MTAP (4/19, 21.1%). Although the number of ampullary tumors was small, KRAS alterations were observed in both cases. Overall, these findings support the concept that BTTs are molecularly heterogeneous according to the primary tumor site. In particular, iCCA was enriched for therapeutically relevant alterations such as FGFR2 fusions/rearrangements and IDH1 mutations, whereas GBC showed enrichment of ERBB2 alterations and eCCA was characterized more frequently by KRAS-, TP53-, and SMAD4-associated molecular patterns. In exploratory comparisons of selected recurrent or clinically relevant alterations, IDH1 alterations were more frequent in iCCA than in non-iCCA tumors (8/46 vs. 0/45; Fisher’s exact test, *p* = 0.006), ERBB2 alterations were more frequent in GBC than in non-GBC tumors (5/24 vs. 0/67; *p* < 0.001), KRAS alterations were more frequent in eCCA than in non-eCCA tumors (9/19 vs. 12/72; *p* = 0.011), and TP53 alterations were more frequent in GBC than in non-GBC tumors (18/24 vs. 33/67; *p* = 0.033). These exploratory comparisons are summarized in Supplementary Table S1 and should be interpreted with caution because they were not adjusted for multiple testing. These site-specific differences suggest that the practical clinical utility of CGP is the greatest in selected molecular subsets, particularly within iCCA. These patterns are illustrated in Figure 1 and Figure 2.

### 3.5. OncoPrint-Style Visualization and Molecularly Informative Cases

An OncoPrint-style figure was generated to provide a compact visual summary of recurrent and clinically relevant genomic alterations across the cohort. The figure displays selected genes, the number of altered genes per patient, and patient-level annotations including primary site, CGP platform, and surgery history. Representative molecularly informative cases in whom genomically matched therapy was introduced are summarized in Table 3.

## 4. Discussion

This updated single-center analysis demonstrated that CGP identified clinically meaningful genomic alterations in a substantial subset of patients with BTTs and revealed clear site-specific differences in their molecular landscape. Although the overall genomic profile of the cohort was dominated by alterations in TP53 and KRAS, genomic findings with more immediate therapeutic relevance were concentrated in selected tumor subtypes. These results support the value of CGP as a tool for molecular characterization, as well as a clinically relevant platform for identifying molecular subsets in which treatment strategies may increasingly diverge from conventional anatomical classification.

The biological heterogeneity of BTTs is a key framework for interpreting these findings. Prior genomic studies have shown that iCCA, eCCA, and GBC harbor distinct genomic profiles. In addition, clinicopathological and molecular studies have demonstrated that iCCA itself is heterogeneous and comprises small duct-type and large duct-type tumors with different biological features. In particular, FGFR2 fusions and IDH1 alterations are enriched in small duct-type iCCA, whereas KRAS-associated molecular patterns are more commonly encountered in large duct-type disease or in non-iCCA biliary tumors. Thus, both anatomical site and histological subtype are important for interpreting the biological and clinical significance of CGP findings [7,8].

The site-specific distribution of genomic alterations observed in this study was biologically plausible and broadly consistent with previous genomic studies of BTTs. In particular, iCCA was enriched for FGFR2 fusions/rearrangements and IDH1 alterations, both of which are well-recognized molecular events in this subtype. In contrast, GBC showed enrichment of ERBB2 alterations, whereas eCCA was more often characterized by KRAS-, TP53-, and SMAD4-associated molecular patterns [1,9,10]. Collectively, these findings reinforce the concept that BTTs should not be regarded as a genomically uniform disease entity and that the clinical utility of CGP should be interpreted in a site-specific context rather than across BTTs as a single category. Importantly, the present study emphasizes the gap between the identification of therapeutically relevant genomic findings and the actual delivery of matched treatment in routine practice.

In our cohort, potentially actionable genomic alterations were identified in 21 patients, expert panel-based therapeutic options (including clinical trials) were identified in 15 patients, and genomically matched therapy was ultimately administered to 7 patients. This stepwise decrease is clinically meaningful; it indicates that the practical value of CGP cannot be judged solely by the presence of potentially actionable alterations or by the expert panel recommendation rate. Rather, real-world benefit depends on whether an appropriate treatment can actually be introduced within the limited therapeutic window available for patients with advanced biliary tract malignancies. Thus, compared with previous studies that primarily described the genomic landscape of BTTs, the present study provides additional real-world information on the clinical pathway from CGP results to expert panel recommendations and actual treatment implementation.

Our experience also underscores the difficulty of translating genomically informed therapeutic options into actual patient benefit. Enrollment in clinical trials for advanced BTTs may be limited by multiple barriers, including geographical inaccessibility of trial sites, strict eligibility criteria, rapid deterioration in general condition, timing of referral for CGP, and competing standard treatment options. Consequently, even when the expert panel identifies a genomically informed therapeutic option, implementation may remain difficult in practice. This issue is particularly important in BTTs, in which disease progression can be rapid and the opportunity to introduce later-line therapy may be narrow.

Moreover, the present results do not indicate limited value of CGP. Rather, they likely reflect the current transitional phase of precision oncology in BTTs. In the present cohort, actual matched treatment was introduced for MSI-H, FGFR2 fusion or rearrangement, ATM alteration, and BRCA2 alteration. In addition, established or emerging therapeutic relevance exists for FGFR2 fusions or rearrangements [11,12], human epidermal growth factor receptor 2-amplified (HER2-amplified) or HER2-expressing tumors [13,14], BRAF V600E [15,16], and IDH1 alterations [17,18]. Although IDH1 mutations were detected in eight patients, no patient received an IDH1 inhibitor in the present cohort, partly because IDH1 inhibitor therapy for BTTs was not approved or reimbursed in Japan during the study period.

Less frequent findings, such as MET amplification and DNA damage repair-related alterations, may also define molecular subsets for which the clinical benefit of CGP is likely to expand [19,20,21,22,23]. From this perspective, the significance of CGP extends beyond currently accessible targets alone and includes the prospective identification of patients who may benefit as biomarker-guided therapeutic options continue to evolve.

KRAS should be interpreted somewhat differently. In the present cohort, KRAS was among the most frequently altered genes overall and was particularly common in eCCA. This distribution was consistent with the results of a large-scale Center for Cancer Genomics and Advanced Therapeutics analysis reporting KRAS mutations in 23.4% of BTTs, with higher frequencies observed in eCCA than in GBC [4]. That study further showed that KRAS mutations, particularly variants such as G12D, were associated with poor prognosis and unfavorable therapeutic outcomes across first-line treatment regimens [4].

Although the immediate therapeutic implications of KRAS alterations remain limited in routine BTT care, rare context-specific settings such as KRAS G12C may become clinically relevant as targeted agents evolve [24]. Therefore, KRAS should be regarded as a biologically informative and clinically stratifying alteration that may contribute to prognostic assessment and therapeutic prediction rather than a major contributor to the immediate therapeutic utility of CGP in everyday BTT care. This distinction is important because recurrent genomic alterations differ in present-day actionability, as well as in their potential value for biological classification and outcome stratification.

Our previous study suggested that actionable gene variants in iCCA were closely linked to small duct-type tumors and to intratumoral vascular penetration on contrast-enhanced ultrasonography. These findings indicate that, even when histological subclassification is limited in non-surgical cases, characteristic imaging features may help identify tumors more likely to harbor druggable variants and may support earlier prioritization of CGP testing [25]. In this context, the present CGP cohort provides complementary real-world evidence that therapeutically relevant findings were the most frequently observed in iCCA but were not limited to this subgroup; ERBB2 alterations in GBC may also represent an important molecular subset as biomarker-guided therapeutic options continue to evolve. The concentration of practical clinical utility in iCCA deserves emphasis. This observation is consistent with current clinical experience showing that FGFR2-rearranged iCCA represents a molecularly defined subset of BTTs with established therapeutic relevance and clinically available FGFR-targeted treatment options.

In our cohort, FGFR2 fusion/rearrangement-positive tumors provided the most direct example in which CGP affected treatment selection in routine care. However, the implementation of pembrolizumab for MSI-H disease and investigational approaches targeting ATM or BRCA2 alterations also illustrates that the clinical impact of CGP is not limited to FGFR2 alone. Accordingly, the immediate clinical yield of CGP appears to be highest in molecular subsets with a biologically validated target, a therapeutically relevant agent or clinical trial, and realistic treatment accessibility. Another important implication of our findings is that the clinical contribution of CGP should be considered at two complementary levels, namely present clinical utility and future therapeutic opportunity. Present clinical utility refers to the ability of CGP to identify alterations that can directly influence treatment selection under current clinical conditions. Future therapeutic opportunity refers to the value of documenting genomic alterations that may become increasingly relevant as new drugs, indications, or clinical trials emerge. In our study, the former was most clearly exemplified by tumors harboring MSI-H, FGFR2 fusions/rearrangements, BRCA2 alteration, or ATM alteration with treatment implementation. In contrast, the latter was represented by alterations that were detected but not implemented as matched treatment in the present cohort, including ERBB2 alterations, IDH1 mutations, BRAF V600E, MET amplification, and other DNA damage repair-related alterations [13,14,15,16,17,18,19,20,21,22,23,26]. This distinction is important because a genomic finding that does not immediately alter treatment in current practice may carry substantial clinical value in the future. These findings are also consistent with previous next-generation sequencing and molecular profiling studies in biliary tract cancers and cholangiocarcinoma, which demonstrated that comprehensive genomic profiling can identify clinically relevant alterations and potential therapeutic targets across anatomical subtypes [27,28]. More broadly, interpretation of CGP results should be aligned with established actionability frameworks and recommendations for clinical NGS implementation, such as ESCAT and the ESMO Precision Medicine Working Group recommendations [29,30]. In addition, practical issues related to assay selection, specimen type, pre-analytical factors, and report interpretation remain important when applying CGP to cholangiocarcinoma in routine clinical practice [31].

This study has several limitations. First, it was a retrospective single-center analysis with a modest sample size, which may limit the generalizability of the findings. In particular, the small number of patients in some tumor-site subgroups, especially eCCA and ampullary tumors, limited the statistical power for site-specific comparisons. In addition, the retrospective single-center design may have introduced selection bias, referral bias, and institutional treatment bias. These factors may have influenced the patient population undergoing CGP, the timing of CGP, expert panel recommendations, access to clinical trials, and the implementation of genomically matched therapy; therefore, the findings may not be fully generalizable to other institutions or healthcare settings.

Therefore, site-specific differences in clinical utility and genomic alteration frequencies should be interpreted as exploratory and descriptive rather than definitive. CGP was performed using multiple platforms, including both tissue-based and liquid-based assays. Assay-specific differences in specimen type, gene coverage, fusion detection, copy number assessment, MSI evaluation, and susceptibility to ctDNA shedding in liquid biopsy may have influenced the observed mutation frequencies and biomarker detection rates. Third, post-CGP treatment courses were not uniformly structured in the clinical dataset, limiting detailed evaluation of treatment implementation in all cases. Fourth, because genomically matched therapy was introduced in only a small number of patients, the present study is more informative regarding the detectability and clinical interpretability of relevant genomic alterations than regarding comparative treatment efficacy. Multivariate analysis was not performed because the number of patients who received genomically matched therapy was small and the number of patients in some tumor-site subgroups was limited, making stable multivariate modeling inappropriate. Because survival outcomes were not evaluated and post-CGP treatment courses were heterogeneous, the present study cannot determine whether CGP or genomically matched therapy improved overall survival or progression-free survival. Nevertheless, the data provide a clinically interpretable overview of the institutional genomic landscape of BTTs and highlight both the practical strengths and the current limitations of CGP in routine care.

## 5. Conclusions

The results of this single-center study suggest that the value of CGP in BTTs lies in identifying the limited number of patients who can currently receive genomically matched therapy and in defining molecular subsets for which the clinical benefit of CGP is likely to expand. In this cohort, potentially actionable genomic alterations were identified in 21 patients, expert panel-based therapeutic options were identified in 15 patients, and genomically matched therapy was introduced in 7 patients. These findings highlighted the clinical value of CGP and the remaining implementation gap in real-world practice. Thus, CGP should be regarded as an important platform for both current clinical decision-making and future therapeutic stratification in BTTs. These findings also suggest the potential importance of considering CGP at a clinically appropriate time point, although the optimal timing of CGP was not systematically evaluated in this study.

## Figures and Tables

**Table 1:** Characteristics of the patients.

Characteristic	N = 91
Age, median (range), years	66 (28–84)
Male sex	52 (57.1%)
**Primary tumor site**	
Intrahepatic cholangiocarcinoma	46 (50.5%)
Extrahepatic cholangiocarcinoma	19 (20.9%)
Gallbladder cancer	24 (26.4%)
Ampullary tumor	2 (2.2%)
**Clinical setting at CGP**	
Postoperative recurrence	27 (29.7%)
Unresectable advanced disease	64 (70.3%)
**Comprehensive genomic profiling platform**	
FoundationOne CDx	36 (39.6%)
NCC Oncopanel	32 (35.2%)
GenMineTOP	10 (11.0%)
FoundationOne Liquid CDx	5 (5.5%)
Guardant360 CDx	8 (8.8%)

**Table 2:** Site-specific clinical utility of comprehensive genomic profiling in biliary tract tumors.

Primary Site	CGP, n	Potentially Actionable Alterations, n (%)	EP Options, n (%)	Matched Therapy, n (%)
iCCA	46	17 (37.0%; 95% CI, 23.2–52.5)	11 (23.9%; 95% CI, 12.6–38.8)	6 (13.0%; 95% CI, 4.9–26.3)
eCCA	19	0 (0.0%; 95% CI, 0.0–17.6)	1 (5.3%; 95% CI, 0.1–26.0)	0 (0.0%; 95% CI, 0.0–17.6)
GBC	24	4 (16.7%; 95% CI, 4.7–37.4)	3 (12.5%; 95% CI, 2.7–32.4)	1 (4.2%; 95% CI, 0.1–21.1)
Ampullary tumor	2	0 (0.0%; 95% CI, 0.0–84.2)	0 (0.0%; 95% CI, 0.0–84.2)	0 (0.0%; 95% CI, 0.0–84.2)
Total	91	21 (23.1%; 95% CI, 14.9–33.1)	15 (16.5%; 95% CI, 9.5–25.7)	7 (7.7%; 95% CI, 3.1–15.2)

Abbreviations: CGP, comprehensive genomic profiling; eCCA, extrahepatic cholangiocarcinoma; EP, expert panel; GBC, gallbladder cancer; iCCA, intrahepatic cholangiocarcinoma.

**Table 3:** Patients who received genomically matched therapy based on CGP results.

Case	Primary Tumor Site	Druggable Biomarker	Treatment Introduced
Case 1	iCCA	MSI-H	Pembrolizumab
Case 2	iCCA	FGFR2-BICC1 fusion	Pemigatinib
Case 3	iCCA	FGFR2-BICC1 fusion	Futibatinib
Case 4	iCCA	FGFR2-BICC1 fusion	Futibatinib
Case 5	iCCA	FGFR2-CCDC147 fusion	Pemigatinib
Case 6	iCCA	ATM T2666A	BAY 1895344, an ATR inhibitor, administered through clinical trial enrollment
Case 7	GBC	BRCA2 mutation	PARP inhibitor administered through clinical trial enrollment

Abbreviations: ATM, ataxia telangiectasia mutated; ATR, ataxia telangiectasia and Rad3-related; BICC1, BicC family RNA binding protein 1; CGP, comprehensive genomic profiling; FGFR2, fibroblast growth factor receptor 2; GBC, gallbladder cancer; iCCA, intrahepatic cholangiocarcinoma; MSI-H, microsatellite instability-high; PARP, poly(ADP-ribose) polymerase.

## Data Availability

The data presented in this study are available from the corresponding author upon reasonable request. The data are not publicly available due to privacy and ethical restrictions.

## Supplementary Materials

The following supporting information can be downloaded at: https://www.mdpi.com/article/10.3390/cancers18142189/s1, Table S1: Exploratory comparisons of selected genomic alterations according to primary tumor site.

## Abbreviations

APC, adenomatous polyposis coli; ARID1A, AT-rich interaction domain 1A; ATM, ataxia telangiectasia mutated; ATR, ataxia telangiectasia and Rad3-related; BICC1, BicC family RNA-binding protein 1; BRAF, B-Raf proto-oncogene, serine/threonine kinase; BRCA1/2, breast cancer gene 1/2; BTT, biliary tract tumor; CDKN2A/B, cyclin-dependent kinase inhibitor 2A/B; CGP, comprehensive genomic profiling; ctDNA, circulating tumor DNA; eCCA, extrahepatic cholangiocarcinoma; EP, expert panel; ERBB2, erb-b2 receptor tyrosine kinase 2; ESCAT, ESMO Scale for Clinical Actionability of Molecular Targets; ESMO, European Society for Medical Oncology; F1CDx, FoundationOne CDx; F1LCDx, FoundationOne Liquid CDx; FGFR2, fibroblast growth factor receptor 2; GBC, gallbladder cancer; HER2, human epidermal growth factor receptor 2; iCCA, intrahepatic cholangiocarcinoma; IDH1, isocitrate dehydrogenase 1; KRAS, KRAS proto-oncogene, GTPase; MDM2, mouse double minute 2 homolog; MET, MET proto-oncogene, receptor tyrosine kinase; MSI-H, microsatellite instability-high; MTAP, methylthioadenosine phosphorylase; NCC Oncopanel, OncoGuide NCC Oncopanel System; NGS, next-generation sequencing; PALB2, partner and localizer of BRCA2; PARP, poly(ADP-ribose) polymerase; PBRM1, polybromo 1; PIK3CA, phosphatidylinositol-4,5-bisphosphate 3-kinase catalytic subunit alpha; SMAD4, SMAD family member 4; TP53, tumor protein p53; UMIN, University Hospital Medical Information Network.

## References

[1] Nakamura H., Arai Y., Totoki Y., Shirota T., Elzawahry A., Kato M., Hama N., Hosoda F., Urushidate T., Ohashi S. (2015). Genomic spectra of biliary tract cancer. Nat. Genet..

[2] Fukada I., Mori S., Hayashi N., Hosonaga M., Wang X., Yamazaki M., Ueki A., Kiyotani K., Tonooka A., Takeuchi K. (2023). Prognostic impact of cancer genomic profile testing for advanced or metastatic solid tumors in clinical practice. Cancer Sci..

[3] Inada H., Miyamoto H., Shinriki S., Oda H., Narahara S., Yoshinari M., Nagaoka K., Yoshii D., Fukubayashi K., Hayashi H. (2024). Clinical utility of a comprehensive genomic profiling test for patient with advanced biliary tract cancer. Int. J. Clin. Oncol..

[4] Iida K., Matsui Y., Urabe Y., Muramatsu T., Matsuzaki J., Saito Y. (2025). Association of KRAS variants with survival and therapeutic outcomes in biliary tract cancers. ESMO Open.

[5] Chen L.T., Vogel A., Hsu C., Chen M.H., Fang W., Pangarsa E.A., Sharma A., Ikeda M., Park J.O., Tan C.K. (2024). Pan-Asian adapted ESMO Clinical Practice Guidelines for the diagnosis, treatment and follow-up of patients with biliary tract cancer. ESMO Open.

[6] Li M.M., Datto M., Duncavage E.J., Kulkarni S., Lindeman N.I., Roy S., Tsimberidou A.M., Vnencak-Jones C.L., Wolff D.J., Younes A. (2017). Standards and guidelines for the interpretation and reporting of sequence variants in cancer: A joint consensus recommendation of the Association for Molecular Pathology, American Society of Clinical Oncology, and College of American Pathologists. J. Mol. Diagn..

[7] Hayashi A., Misumi K., Shibahara J., Arita J., Sakamoto Y., Hasegawa K., Kokudo N., Fukayama M. (2016). Distinct clinicopathologic and genetic features of 2 histologic subtypes of intrahepatic cholangiocarcinoma. Am. J. Surg. Pathol..

[8] Komuta M. (2022). Intrahepatic cholangiocarcinoma: Tumour heterogeneity and its clinical relevance. Clin. Mol. Hepatol..

[9] Sicklick J.K., Fanta P.T., Shimabukuro K., Kurzrock R. (2016). Genomics of gallbladder cancer: The case for biomarker-driven clinical trial design. Cancer Metastasis Rev..

[10] Tsilimigras D.I., Stecko H., Moris D., Pawlik T.M. (2025). Genomic profiling of biliary tract cancers: Comprehensive assessment of anatomic and geographic heterogeneity, co-alterations and outcomes. J. Surg. Oncol..

[11] Abou-Alfa G.K., Sahai V., Hollebecque A., Vaccaro G., Melisi D., Al-Rajabi R., Paulson A.S., Borad M.J., Gallinson D., Murphy A.G. (2020). Pemigatinib for previously treated, locally advanced or metastatic cholangiocarcinoma: A multicentre, open-label, phase 2 study. Lancet Oncol..

[12] Goyal L., Meric-Bernstam F., Hollebecque A., Valle J.W., Morizane C., Karasic T.B., Abrams T.A., Furuse J., Kelley R.K., Cassier P.A. (2023). Futibatinib for FGFR2-rearranged intrahepatic cholangiocarcinoma. N. Engl. J. Med..

[13] Harding J.J., Fan J., Oh D.Y., Choi H.J., Kim J.W., Chang H.M., Bao L., Sun H.C., Macarulla T., Xie F. (2023). Zanidatamab for HER2-amplified, unresectable, locally advanced or metastatic biliary tract cancer (HERIZON-BTC-01): A multicentre, single-arm, phase 2b study. Lancet Oncol..

[14] Ohba A., Morizane C., Kawamoto Y., Komatsu Y., Ueno M., Kobayashi S., Ikeda M., Sasaki M., Furuse J., Okano N. (2024). Trastuzumab deruxtecan in human epidermal growth factor receptor 2-expressing biliary tract cancer (HERB; NCCH1805): A multicenter, single-arm, phase II trial. J. Clin. Oncol..

[15] Subbiah V., Lassen U., Élez E., Italiano A., Curigliano G., Javle M., de Braud F., Prager G.W., Greil R., Stein A. (2020). Dabrafenib plus trametinib in patients with BRAF(V600E)-mutated biliary tract cancer (ROAR): A phase 2, open-label, single-arm, multicentre basket trial. Lancet Oncol..

[16] Tang T.Y., Nichetti F., Kaplan B., Lonardi S., Pietrantonio F., Salvatore L., Vivaldi C., Rimassa L., de Braud F., Rizzato M.D. (2023). Comparative genomic analysis and clinical outcomes of BRAF-mutated advanced biliary tract cancers. Clin. Cancer Res..

[17] Abou-Alfa G.K., Macarulla T., Javle M.M., Kelley R.K., Lubner S.J., Adeva J., Cleary J.M., Catenacci D.V., Borad M.J., Bridgewater J. (2020). Ivosidenib in IDH1-mutant, chemotherapy-refractory cholangiocarcinoma (ClarIDHy): A multicentre, randomised, double-blind, placebo-controlled, phase 3 study. Lancet Oncol..

[18] Zhu A.X., Macarulla T., Javle M.M., Kelley R.K., Lubner S.J., Adeva J., Cleary J.M., Catenacci D.V., Borad M.J., Bridgewater J. (2021). Final overall survival efficacy results of ivosidenib for patients with advanced cholangiocarcinoma with IDH1 mutation: The phase 3 randomized clinical ClarIDHy trial. JAMA Oncol..

[19] Kang E.J., Yang Y., Lee S., Kim Y.J., Lim S.M., Ahn M.J., Choi Y.J., Lee Y., Kim T.M., Kim I. (2024). A phase II study of tepotinib in patients with advanced solid cancers harboring MET exon 14 skipping mutations or amplification (KCSG AL19-17). ESMO Open.

[20] Mavroeidi I.A., Burghofer J., Kalbourtzis S., Taghizadeh H., Webersinke G., Piringer G., Kasper S., Schreil G., Liffers S.T., Reichinger A. (2024). Understanding homologous recombination repair deficiency in biliary tract cancers: Clinical implications and correlation with platinum sensitivity. ESMO Open.

[21] Sadagopan N., Wang H., Yin C., Weinberg B.A., Noel M.S., Mukherji R., Geng X., Marshall J.L., He A.R. (2025). A phase II single-arm study of combination pembrolizumab and olaparib in the treatment of patients with advanced biliary tract cancer. npj Precis. Oncol..

[22] Speckart J., Rasmusen V., Talib Z., GnanaDev D.A., Rahnemai-Azar A.A. (2024). Emerging therapies in management of cholangiocarcinoma. Cancers.

[23] Yamada D., Kobayashi S., Doki Y., Eguchi H. (2025). Genomic landscape of biliary tract cancer and corresponding targeted treatment strategies. Int. J. Clin. Oncol..

[24] Bekaii-Saab T.S., Yaeger R., Spira A.I., Pelster M.S., Sabari J.K., Hafez N., Barve M., Velastegui K., Yan X., Shetty A. (2023). Adagrasib in advanced solid tumors harboring a KRAS(G12C) mutation. J. Clin. Oncol..

[25] Nakaoka K., Ohno E., Yamada S., Kuzuya T., Sudo T., Ueno S., Tanaka H., Sasaki Y., Miyahara R., Hashimoto S. (2025). Contrast-enhanced ultrasound imaging characteristics of intrahepatic cholangiocarcinoma with actionable gene variants detected by comprehensive cancer gene panel testing. Int. J. Clin. Oncol..

[26] Subbiah V., Kreitman R.J., Wainberg Z.A., Gazzah A., Lassen U., Stein A., Wen P.Y., Dietrich S., de Jonge M.J.A., Blay J.Y. (2023). Dabrafenib plus trametinib in BRAFV600E-mutated rare cancers: The phase 2 ROAR trial. Nat. Med..

[27] Lowery M.A., Ptashkin R., Jordan E., Berger M.F., Zehir A., Capanu M., Kemeny N.E., O’Reilly E.M., El-Dika I., Jarnagin W.R. (2018). Comprehensive molecular profiling of intrahepatic and extrahepatic cholangiocarcinomas: Potential targets for intervention. Clin. Cancer Res..

[28] Javle M., Bekaii-Saab T., Jain A., Wang Y., Kelley R.K., Wang K., Kang H.C., Catenacci D., Ali S., Krishnan S. (2016). Biliary cancer: Utility of next-generation sequencing for clinical management. Cancer.

[29] Mosele F., Remon J., Mateo J., Westphalen C.B., Barlesi F., Lolkema M., Normanno N., Scarpa A., Robson M., Meric-Bernstam F. (2020). Recommendations for the use of next-generation sequencing (NGS) for patients with metastatic cancers: A report from the ESMO Precision Medicine Working Group. Ann. Oncol..

[30] Mateo J., Chakravarty D., Dienstmann R., Jezdic S., Gonzalez-Perez A., Lopez-Bigas N., Ng C.K.Y., Bedard P.L., Tortora G., Douillard J.Y. (2018). A framework to rank genomic alterations as targets for cancer precision medicine: The ESMO Scale for Clinical Actionability of molecular Targets (ESCAT). Ann. Oncol..

[31] Stenzinger A., Vogel A., Lehmann U., Lamarca A., Hofman P., Terracciano L., Normanno N. (2024). Molecular profiling in cholangiocarcinoma: A practical guide to next-generation sequencing. Cancer Treat. Rev..

