# Supplementary material for: Clinical Utility and Genomic Landscape of Comprehensive Genomic Profiling in Biliary Tract Tumors: A Single-Center Real-World Study
Source: Cancers (Basel). 2026 Jul 8;18(14):2189. doi: 10.3390/cancers18142189 (PMC13406710; doi:10.3390/cancers18142189)
Supplement: Supplementary file 1 [file cancers-18-02189-s001.zip › cancers-4406836-supplementary.pdf]

Table S1. Exploratory comparisons of selected genomic alterations according to primary tumor site.

Genomic alteration	Comparison	Tumor-site subgroup, n/N (%)	Comparator, n/N (%)	p value
IDH1 alteration	iCCA vs. non-iCCA	8/46 (17.4%)	0/45 (0.0%)	0.006
ERBB2 alteration	GBC vs. non-GBC	5/24 (20.8%)	0/67 (0.0%)	<0.001
KRAS alteration	eCCA vs. non-eCCA	9/19 (47.4%)	12/72 (16.7%)	0.011
TP53 alteration	GBC vs. non-GBC	18/24 (75.0%)	33/67 (49.3%)	0.033

Abbreviations: eCCA, extrahepatic cholangiocarcinoma; GBC, gallbladder cancer; iCCA, intrahepatic cholangiocarcinoma.
p values were calculated using Fisher's exact test.
